# Long COVID-19/post-COVID condition in children: do we all speak the same language?

**DOI:** 10.1186/s13052-023-01417-8

**Published:** 2023-01-20

**Authors:** Silvia Garazzino, Marco Denina, Giulia Pruccoli, Elisa Funiciello, Ugo Ramenghi, Franca Fagioli

**Affiliations:** 1 Unit of Pediatric Infectious Diseases, University of Turin, Regina Margherita Children’s Hospital, Turin, Italy; 2 Pediatric Emergency Department, Regina Margherita Children’s Hospital, Turin, Italy; 3 Department of Public Health and Pediatric Sciences, University of Turin, Regina Margherita Children’s Hospital, Turin, Italy

**Keywords:** SARS-CoV-2 infection, Children, Long-COVID-19, Post-COVID condition

## Abstract

Post-COVID condition is a new and highly debated entity that is still to be outlined in its complexity, especially in the pediatric population. In response to the article by Trapani and colleagues, we report the results of a long-term follow-up conducted in the outpatient clinic of the Pediatric Infectious Diseases Unit on children admitted to our hospital with SARS-CoV-2 infection.

Dear Editor,

We read with interest the article by Trapani et al. on Long COVID-19 in children [1]. Authors presented data obtained from Italian General Practitioners (GPs) through questionnaires addressed to children in primary care, including those residents in Piedmont, Italy, and some hospitalized children with COVID-19. The reported cumulative incidence of Long COVID-19 symptoms in 60 hospitalized children was 58.3%, much higher than what we observed in our reality. At Regina Margherita Children's Hospital (Turin, Italy), which is the referral pediatric hospital for Piedmont region, more than 500 children with SARS-CoV-2 infection have been hospitalized since the beginning of the pandemic up to January 2022. We hereby report the results of a long-term follow-up conducted in the outpatient clinic of the Pediatric Infectious Diseases Unit on children admitted to our hospital with SARS-CoV-2 infection. A subset of children, including those with moderate to severe COVID-19, chronic comorbidities or presenting sequelae at discharge, underwent a strict follow-up consisting in periodic clinical assessments, laboratory and instrumental evaluations. The remaining children were followed-up throughout telephonic contacts and, if necessary, through clinical assessments and targeted investigations to rule out post-COVID conditions (PCC). Patients with recently diagnosed oncoematologic diseases and children with preexisting severe neurologic impairment were excluded from the present analysis.

A total of 417 infants and children with a median age 3.64 years (Inter Quartile Range –IQR: 0.5-10.34) were evaluated: 243 (58,3%) were males and 147 (35.2%) had concomitant chronic diseases. Mean hospital stay was 8 days, median 5 days (IQR 3 – 9). A complicated course of SARS-CoV-2 infection was observed in 140 children (33%): among these, 52 had pneumonia and 22 required intensive care admission.

Mean duration of follow-up was 9.8 months (range 6 – 18 months). At 4 and 12 weeks from SARS-CoV-2 infections 35 children (8.4%) presented at least one symptom that fulfilled the updated criteria for PCC in children and young people [2]: persistent asthenia and easy fatigue in 13 children, residual respiratory distress under exertion in 11, neurological alterations in fine motor skills, dizziness and problems in equilibrium maintenance in 8, frequent arthromyalgies and persistent lack of appetite in 4 children each, and hemicranias, cardiac disturbancies (heart-pounding), chest pain, cough and wheezing in 2 children each.

At 6 months from infection 25 children still had PCC symptoms; none developed new PCC-related manifestations after 3 months from the infection. Children with PCC were significantly older than those without (mean age 9.87 years vs 5.32, respectively; p <0.0001).

No patient required a second hospital admission for reasons related to SARS-CoV-2 infection, including PCC.

PCC is a new and highly debated entity that is still to be outlined in its complexity, especially in the pediatric population. In the latest months, a rapidly increasing number of studies has been published, investigating the frequency, characteristics, risk factors and possible underlying mechanisms of such condition in adults, but also in children and young people. In adults, PCC appears unrelated to COVID-19 severity and may be driven by long-term tissue damage, following persistent viral-induced inflammation, immune dysregulation, autoimmune phenomena, endothelial damage and micro thrombosis. Risk factors for PCC identified in the adult population include female sex, more than five symptoms at onset, early dyspnoea, preexisting psychiatric disorders and increased inflammatory biomarkers [3]. In children and adolescents similar pathogenetic mechanisms are plausible, but also vitamin and micronutrients deficiencies have been postulated [4]. Suggested risk factors for PCC in the pediatric population include older age (especially > 12 years), muscle pain at COVID-19 onset, allergic diseases and ICU admission; potential treatments are rehabilitation and symptomatic drugs as for adults, dietary precautions and vitamin supplementation [4–6].

Although the possibility of long-term consequences of COVID-19 is universally recognized, multiple methodological issues on available data have been raised, including a lack of harmonization in definitions and terminology, and the absence of standardized clinical outcomes to be routinely assessed through objectivable measures [7]. Such heterogeneity into research on PCC makes results hardly comparable.

Precisely due to the wide variability of prevalence estimates for PCC in children and young people (ranging from 1% to 51% ), Stephenson et al. derived a research definition aligned to the clinical World Health Organization (WHO) case definition, to allow comparisons between research studies as follows: *Post-COVID-19 condition occurs in young people with a history of confirmed SARS-CoV-2 infection, with at least one persisting physical symptom for a minimum duration of 12 weeks after initial testing that cannot be explained by an alternative diagnosis. The symptoms have an impact on everyday functioning, may continue or develop after COVID infection, and may fluctuate or relapse over time* [2].

An international Delphi consensus study identified the core outcome set (COS) for PCC in adults, including eleven items: fatigue, pain, post-exertion symptoms, work or occupational and study changes, survival, functioning, symptoms and conditions for each of cardiovascular, respiratory, nervous system, cognitive, mental health, and physical outcomes [8].

In children and young adults there is an urgent need to develop a COS, preferably structured for different age groups, with age-appropriate measurements and data harmonization processing tools for research on PCC.

Self-completed questionnaires administered to families and not associated with targeted assessments (especially from a neurologic or psychiatric perspective) and not supported by a medical examination risk to overestimate PCC. Indeed, many reported symptoms in older children and adolescents (such as fatigue and psychological symptoms) may be related to the complex social situation that occurred during the Pandemic as a consequence of school closure, suspension of recreational activities and social isolation. In our opinion, children identified by an indirect approach (phone call, parental questionnaires) as suffering from PCC should be clinically assessed by a multidisciplinary team to confirm or rule out the diagnosis and schedule an appropriate follow-up.

In a nationwide cohort study, Boch et al. demonstrated that symptoms lasting > 4 weeks reported through an electronic questionnaire were common also in control children, with an estimated PCC prevalence of 0.8% in the SARS-CoV-2 positive group. Authors concluded that concentration difficulties, headache, muscle and joint pain as well as nausea should not be considered symptoms of PCC as they were frequently reported also in the control group. On the contrary, fatigue, loss of smell and taste, dizziness, muscle weakness, chest pain and respiratory problems were more frequently found in SARS-CoV-2 children, especially in those aging 6-17 years and were thus not attributed to psychological sequelae of social restrictions [9].

In our cohort of hospitalized children, with a minimum 6 month-follow-up, PCC was rare, also considering that many patients had pre-existing concomitant diseases. Only symptoms occurring or persisting within 12 weeks from SARS-CoV-2 infection were deemed consistent with sequelae of COVID-19, according to temporal criteria established by WHO in adults [10]. Furthermore, a long recovery is relatively common in other viral diseases, such as EBV, CMV or enteric viruses infections and a short follow-up of SARS-CoV-2 infected children could be misleading.

The type of the population investigated, composed exclusively by inpatients, might be a limit of our study and could lead to PCC overestimates: although we also included asymptomatic infected children who were admitted for reasons other than COVID-19 (i.e. traumatic injuries), our cohort might not be fully representative of the general pediatric population due the high number of chronic patients, potentially more prone to PCC. Another limit of our report is the lack of a non-infected control group.

In conclusion, PCC in the pediatric population is still an ill-defined entity that urgently needs agreed and standardized definitions, as well as outcome measures. Only when these items are conclusively established, the real prevalence of the disease will be clarified.

## Data Availability

The datasets used and/or analysed during the current study are available from the corresponding author on reasonable request but restrictions apply to the availability of these data due to privacy protection rules.

## Abbreviations

PCC: Post-COVID condition
ICU: Intensive care unit
COS: Core outcome set
IQR: InterQuartile Range
WHO: World Health Organization
